# Angiotensin II Receptor Blockers (ARBs Antihypertensive Agents) Increase Replication of SARS-CoV-2 in Vero E6 Cells

**DOI:** 10.3389/fcimb.2021.639177

**Published:** 2021-06-11

**Authors:** Gabriel Augusto Pires de Souza, Ikram Omar Osman, Marion Le Bideau, Jean-Pierre Baudoin, Rita Jaafar, Christian Devaux, Bernard La Scola

**Affiliations:** ^1^ Aix-Marseille Université, Institut de Recherche pour le Développement (IRD), Assistance Publique - Hôpitaux de Marseille (AP-HM), MEPHI, Marseille, France; ^2^ Microbes, Evolution, Phylogénie et Infection (MEPHI), IHU - Méditerranée Infection, Marseille, France; ^3^ Centre National de La Recherche Scientifique (CNRS), Marseille, France

**Keywords:** SARS-CoV-2, COVID-19, angiotensin II receptor blockers, antihypertensive, angiotensin I converting enzyme 2, Vero E6 cells

## Abstract

Several comorbidities, including hypertension, have been associated with an increased risk of developing severe disease during SARS-CoV-2 infection. Angiotensin II receptor blockers (ARBs) are currently some of the most widely-used drugs to control blood pressure by acting on the angiotensin II type 1 receptor (AT1R). ARBs have been reported to trigger the modulation of the angiotensin I converting enzyme 2 (ACE2), the receptor used by the virus to penetrate susceptible cells, raising concern that such treatments may promote virus capture and increase their viral load in patients receiving ARBs therapy. In this *in vitro* study, we reviewed the effect of ARBs on ACE2 and AT1R expression and investigated whether treatment of permissive ACE2+/AT1R+ Vero E6 cells with ARBs alters SARS-CoV-2 replication *in vitro* in an angiotensin II-free system. After treating the cells with the ARBs, we observed an approximate 50% relative increase in SARS-CoV-2 production in infected Vero E6 cells that correlates with the ARBs-induced up-regulation of ACE2 expression. From this data, we believe that the use of ARBs in hypertensive patients infected by SARS-CoV-2 should be carefully evaluated.

## Introduction

The emergence and rapid spread of severe acute respiratory syndrome (SARS) in patients hospitalised in China in late 2019 led to the discovery of a novel coronavirus (Huang et al., 2020; Li et al., 2020; Zhou et al., 2020). The SARS-coronavirus 2 (SARS-CoV-2), which later spread across the globe and reached pandemic status, has forced several countries to adopt lockdown measures, under the threat of the health system collapsing, despite the fact that this put the economy at risk (Shang et al., 2020). Almost one-and-a-half years after the first outbreak of coronavirus disease 2019 (COVID-19) in China, the disease has caused more than three million deaths for 145 million people infected worldwide as of 25 April 2020 (https://coronavirus.jhu.edu/map.html).

SARS-CoV-2 represents one of the most severe threats to human health in more than a century. Previously, other beta coronaviruses caused outbreaks of historical significance, such as the SARS-coronavirus and the Middle East respiratory syndrome (MERS) (Drosten et al., 2003; Zaki et al., 2012; Chakravarty et al., 2020; Zhou et al., 2020). However, despite the previous experience of the SARS-CoV outbreak, much remains uncertain about SARS-CoV-2 infection and coronavirus disease 2019 (COVID-19). Some risk groups have been identified as being more likely to develop severe manifestations of the disease, such as patients with as an underlying lung injury (Yang et al., 2020). Individuals with pre-existing lung disease, diabetes mellitus, and hypertension are at particular risk for SARS-CoV-2 infection (Chen et al., 2020; Liu et al., 2020).

Hypertension is associated with the renin-angiotensin system (RAS), a cascade of vasoactive peptides that orchestrate key human physiology (Wu et al., 2020). The ACE2 monocarboxypeptidase plays a key role in this cascade by acting as a regulator of blood pressure homeostasis through its ability to catalyse the proteolysis of Angiotensin II (AngII) into Ang (Drosten et al., 2003; Zaki et al., 2012; Chakravarty et al., 2020; Huang et al., 2020; Li et al., 2020; Shang et al., 2020; Zhou et al., 2020, Wu et al., 2020). Deregulation of the RAS pathway leads to an increase in the level of AngII, which interacts with its angiotensin II type 1 receptor (AT1R), thus facilitating the activation of various intracellular pathways involved in the remodelling of the heart, vascular disease and endothelial dysfunction (Muslin, 2008; Wehbe et al., 2020).

Interestingly, ACE2 is used by SARS-CoV-2 as a receptor for cell adhesion, which is the initial stage of the virus multiplication cycle (Mostafa-Hedeab, 2020; Shang et al., 2020; Zhou et al., 2020). Once the virus finds the receptor of a permissive cell, it will penetrate it and control the cellular machinery for its replication. As ACE2 is critical to SARS-CoV-2 infection, an imbalance in the RAS, with a shift towards ACE/Ang II and suppressing ACE2/Ang- (Drosten et al., 2003; Zaki et al., 2012; Chakravarty et al., 2020; Huang et al., 2020; Li et al., 2020; Shang et al., 2020; Zhou et al., 2020), may be an important mediator of COVID-19 pathophysiology (South et al., 2020). However, many individuals with hypertension are prescribed Angiotensin II receptor blockers (ARBs) as a drug to control their blood pressure.

To date, however, there has been much debate about the relationship between the use of angiotensin-converting enzyme inhibitors (ACEi) and ARBs and the risk associated with COVID-19 (Devaux, 2020; Sama et al., 2020; Sriram and Insel, 2020; Zhang et al., 2020). Some authors suspected that treating hypertension and other cardiovascular diseases with antihypertensive drugs could increase ACE2 levels and increase the risk of SARS-CoV-2 infection and/or replication, leading to an increased risk of admission to the intensive care unit, mechanical ventilation, and, sometimes, death in patients with COVID-19 (Diaz, 2020; Fang et al., 2020; Ferrario et al., 2020). Nevertheless, in general, it is understood that there is still a long way to go to reach any conclusions in this regard (Zheng et al., 2020). In this study, we investigated whether the previous treatment of SARS-CoV-2 permissive cells with different ARBs modulates the expression of AT1R and ACE2 and impacts the virus replication in a system without Ang II.

## Materials And Methods

### Cell Line Culture

The Vero E6 cell line (American type culture collection ATCC^®^ CRL-1586 ™) were cultured in minimum essential medium (MEM. Gibco, Thermo Fischer) containing 4% foetal bovine serum (FBS. Invitrogen) and 1% l_glutamine (L-Gln. Invitrogen) at 37°C in a 5% CO_2_ atmosphere using a 175 cm^2^ flasks. Every two days, the medium was replenished, and the confluent culture flask was subcultured by trypsinisation.

Callu-3 cells (ATCC^®^ HTB-55 ™) were in turn cultured in MEM containing 10% FBS and 1% L-Gln in 175 cm^2^ flasks. In tests using culture plaques, the cells were prepared three days earlier and were incubated at 37°C and 5% CO_2_. Caco-2 cells (ATCC^®^ HTB-37 ™) were also cultured in 175 cm^2^ flasks at 37°C and 5% CO_2_, using DMEM medium supplemented with 10% FBS, 1% L-Gln, and 1% amino acids (Aa).

### Drugs

Azilsartan, Eprosartan mesylate, Irbesartan, Losartan potassium, Olmesartan medoxomil, Telmisartan, and Valsartan were purchased from the Sigma Group. All ARBs were pre-diluted in DMSO according to the manufacturer’s instructions.

### Virus Production and Titration

The SARS-CoV-2 (strain IHUMI-3) was previously isolated from the liquid collected from a nasopharyngeal swab (Gautret et al., 2020). The isolate passed through a total of four passages in Vero E6 cells) in culture medium supplemented with 4% FBS and 1% L-Gln, incubated at 37°C in a 5% CO_2_ atmosphere. Following the fourth passage in monolayers of Vero E6 cells grown in 75 cm^2^ flasks, and with the almost complete cytopathic effect (about 72 hours after infection), the supernatant was collected, centrifuged at 3,000×g for 10 minutes at 4°C and then filtered through a 0.22 µm membrane. The filtrate was supplemented with 10% FBS and 1% 2- [4- (2-hydroxyethyl) piperazin-1-yl] ethanesulfonic acid (HEPES) buffer and stored at -80°C, making up the viral stock of SARS-CoV- 2. The 50% tissue culture infectious dose of the virus (TCID50) was determined using 6×10^5^ Vero E6 cells/well in 96 cell plates, using eight replicate wells by dilution. The TCID50 was calculated according to the Spearman and Kärber algorithm.

### Cell Viability Assay

*In vitro* cell viability evaluations were performed on the Vero E6, Calu-3, and Caco-2 cell lines according to the method described by Mossman, with slight modifications (Mosmann, 1983). In a 96-well plate, 6×10^5^ cells were incubated, and 200 µL of the Azilsartan, Eprosartan, Irbesartan, Losartan, Olmesartan, Telmisartan, or Valsartan at different concentrations (1 mM, 500 µM, 250 µM, 125 µM, 62.5 µM, 31.25 µM, 15.62 µM, and 7.81 µM) was added to the wells, diluted in culture medium (MEM 4% FBS and 1% L-Gln for Vero E6, MEM 10% FBS and 1% L-Gln for Calu-3 and DMEM 10% FBS plus 1% Aa and 1% L-Gln for Caco-2), and incubated at 37°C in a 5% CO_2_ atmosphere. Following incubation, the supernatant from each well was replaced by 100 µl of culture medium, supplemented with 10 µl of MTT solution (3-(4,5-dimethyl-2-thiazolyl)-2,5-diphenyl-2H-tetrazolium bromide, Sigma Aldrich, France) (5 mg/ml in PBS), followed by a four-hour incubation at 37°C in a 5% CO_2_ atmosphere. To dissolve the formazen crystals formed by the living cells, 50µL DMSO was added to each well and incubated for 30 minutes at 37°C in a 5% CO_2_ atmosphere. Absorbance was measured at 540 nm using a TECAN Infinite F200 microplate reader. The non-cytotoxic concentration was evaluated based on the percentage of viable cells compared to non-treated cell control. Concentrations that exhibited more than 90% of viable cells after five days were considered non-cytotoxic.

### Ribonucleic Acid (RNA) Extraction and Quantitative-Reverse Transcription Polymerase Chain Reaction (qRT-PCR)

24-well plates were prepared with 2×10^5^ cells/well of Vero E6, using MEM with 4% of FBS and 1% L-Gln. They were incubated again overnight at 37°C in a 5% CO_2_ atmosphere. Cell culture supernatant was removed and replaced by drugs diluted in the culture medium. The drug concentrations tested were those previously defined as non-cytotoxic by MTT assay. Following the treatment 72 hours, the cells were infected with SARS-CoV-2 at a multiplicity of infection (MOI) of 0.1 for 24 hours at 37°C in a 5% CO_2_ incubator. In accordance with the manufacturer’s instructions, RNAs were extracted from cells using a RNeasy Mini Kit (QIAGEN SA) with a DNase I step to eliminate DNA contaminants. The quantity and quality of the RNA was evaluated using a Nanodrop 1000 spectrophotometer (Thermo Science). The first-strand cDNA was obtained using oligo(dT) primers and Moloney murine leukaemia virus reverse transcriptase (MMLV-RT kit; Life Technologies), using 100 ng of purified RNA. The qPCR experiments were performed using specific oligonucleotide primers and hot-start polymerase (SYBR Green Fast Master Mix; Roche Diagnostics). The amplification cycles were performed using a C1000 Touch Thermal cycler (Biorad). The specific primers used were Angiotensin Convertase Enzyme (ACE2) primers (Fwd: 5’ CAG AGG GTG AAC ATA CAG TTG G 3’; Rev: 5’ CAG GGA ACA GGT AGA GGA CAT 3’) and Angiotensin receptor 1 (AT1R) (Fwd: 5’ TGTGGACTGAACCGACTTTTCT 3’; Rev: 5’ GGAACTCTCATCTCCTGTTGCT 3’). The results of qRT-PCR were normalised using the housekeeping gene β-Actin (ACTB) (Fwd: 5’ CAT GCC ATC CTG CGT CTG GA 3’; Rev: 5’ CCG TGG CCA TCT CTT GCT CG 3’) and expressed as relative expression (2^-ΔCT^), where ΔCT = CT (Target gene) – CT (Actin).

### Western Immunoblotting Assay

Vero E6 (2x105 cells/well) were cultured in flat-bottom 24-well plates for 12 hours. Cell culture supernatant was removed and replaced by drugs diluted in the culture medium. After 72 hours of treatment, the cells were then infected with SARS-CoV-2 at a multiplicity of infection (MOI) of 0.01 for 24 hours at 37°C in a 5% CO_2_ atmosphere. Following infection, the cells were immediately washed with ice-cold phosphate-buffered saline (PBS) and lysed on the plate in a 1X RIPA buffer [100 mM Tris-HCl pH7.5; 750 mM NaCl; 5mM EDTA; 5% Igepal, 0.5% sodium dodecyl sulfate (SDS); 2.5% Na Deoxycholate] supplemented with a protease cocktail inhibitor and phosphatase inhibitor (Roche, Germany). 100 µg of protein was loaded onto 10% SDS polyacrylamide gels. After being transferred onto a Nitrocellulose membrane, the blots were incubated for one hour at 4°C with a blocking solution [5% Free Fat Milk (FFM)-1XPBS-0.3% Tween 20], followed by overnight incubation with a goat ACE2 polyclonal antibody (1:1,000, MAB933, R&D Systems, Minneapolis, USA). After three washes in 1XPBS-0,3%Tween 20, the membrane was incubated with a Donkey anti-Goat IgG Horseradish peroxidase-conjugated (1:10,000 dilution with a blocking solution) for two hours at room temperature. A mouse Glyceradehyde-3-Phosphate dehydrogenase (GADPH) monoclonal antibody (1:5,000, Abnova, Taiwan), followed by incubation with a Sheep anti-mouse Horseradish peroxidase-conjugated (1:10,000 dilution with a blocking solution) (Life Technologies, France) as the loading control. The proteins were visualized using an ECL Western Blotting Substrate (Promega, USA), and images were digitized using a Fusion FX (Vilber Lourmat, France). As for the spike expression, the JessTM Simple Western system of automated Western immunoblotting was used, as previously described (Edouard et al., 2020).

### Flow Cytometry

Flow cytometry was used to study the expression of ACE2 on the surface of cells. To do so, Vero E6 cells were cultured in flat-bottom six-well plates at an initial concentration of 1×10^6^ cells/well and were then treated with the drugs. Cells were then detached from the solid support using 2 mM of EDTA in PBS with an incubation period of 15 minutes at 4°C, followed by centrifugation of 500 g for five minutes. The pellet was resuspended in a saturation buffer (2mM EDTA, 10% FSB in PBS) for 30 minutes. For ACE2 labelling, an anti-ACE2-PE mAb (R&D Systems, Minneapolis, USA) was incubated with cells at 4°C in the dark for 30 minutes. Fluorescence intensity was measured using a Canto II cytofluorometer (Becton Dickinson, Biosciences, Le Pont de Claix, France). The results were further analysed using a BD FACSDiva Software v.6.1.3 (Becton, Dickinson and Company, New Jersey, USA).

### Immunofluorescence Assay

Vero E6 cells were cultured on sterile coverslips in 24-well plates at an initial concentration of 2×10^5^ cells/mL. After 72 hours of treatment with the drugs, the cells were then infected with the SARS-CoV-2 at a multiplicity of infection (MOI) of 0.01 for 24 hours at 37°C in a 5% CO2 atmosphere. Following infection, the cells were fixed with paraformaldehyde (4%), permeabilised with 0.1% Triton X-100 for three minutes, and saturated with 3%BSA-0,1% Tween 20-PBS for 30 minutes at room temperature. For the primary labelling, a goat ACE2 polyclonal antibody (1:1,000, MAB933, R&D Systems, Minneapolis, USA), a mouse AT1R monoclonal antibody (1:1,000, MAB102441, R&D Systems, Minneapolis, USA), and a polyclonal rabbit anti-SARS Coronavirus Spike protein (rabbit) (1:1,000, PA1-41142, Thermo Fisher, France.) were used. The 4’,6’-diamino-2-fenil-indol (DAPI) (1:25,000, Life Technologies) and the Phalloidin (Alexa 488) (1:500, OZYME) were used for respectively staining the nucleus and the filamentous actin. After one hour of incubation, the slide was washed with 1XPBS-0,1% Tween 20 and incubated for 30 minutes at room temperature with a mix of Donkey anti-Goat IgG (H+L) Secondary Antibody (Alexa Fluor 555), Goat anti-Rabbit IgG (H+L) Secondary Antibody (Alexa Fluor 647) and Goat anti-Mouse IgGb (H+L) Secondary antibody (Alexa Fluor 488) (1:1,000, Thermo Fischer Scientific). The fluorescence was analysed using laser scanning confocal microscopy. Images were acquired using a confocal microscope (Zeiss LSM 800) with a 63X/1.4 oil objective, an electronic magnification of 0.5, and a resolution of 1014_1014 pixels.

### Drug Testing Procedure

We prepared 96-well plates with 10^6^ cells/well of Vero E6, cultured in MEM with 4% FBS and 1% L-Gln. They were incubated overnight at 37°C in a 5% CO_2_ atmosphere. Cell culture supernatant was removed and replaced by drugs diluted in the culture medium. The drug concentrations tested were those previously defined as non-cytotoxic by MTT assay. After the drugs had been incubated with the cells for 72 hours, the virus suspension (SARS-CoV-2) in culture medium was added to all wells, except the negative controls (where 50 μL of the medium was added), respecting a multiplicity of infection (MOI) of 0.004. The supernatant (t=0) was immediately collected (100 µL) and 24- and 48-hours post-infection (h.p.i.), both were stored at -80°C until RNA extraction. 100 μL from each well was then collected and added to 100 μL of the ready-use VXL buffer from the QIAcube kit (Qiagen, Germany). Extraction was performed using the manual High Pure RNA Isolation Kit (Roche Life Science), following the recommended procedures. The RT-PCR was performed using the Roche RealTime PCR Ready RNA Virus Master Kit. The primers were designed against the N gene (Fwd: 5’ GACCCCAAAATCAGCGAAAT 3’; Rev: 5’ TCTGGTTACTGCCAGTTGAATCTG 3’) using the Roche LightCycler^®^ 480 Instrument II. Relative viral quantification was performed compared to the untreated control (viruses without drugs) using the 2^(–ΔCT)^ method, where ΔCt = {[(Ct 48 h.p.i) treated well] – [mean (Ct 0 h.p.i)]} (Livak and Schmittgen, 2001). We performed a statistical analysis using GraphPad Prism v9.0.0 (GraphPad Software, La Jolla, California USA). The distribution of the data did not follow a normal law. Therefore, a non-parametric Kruskal-Wallis test was used to compare each combination against positive controls using ΔCt between t48 h.p.i. and t0 h.p.i. Finally, Dunn’s test was used to correct multiple comparisons. All tests were used at p = 0.05 parameter and were bilateral (two-sides). From the supernatant collected 24 h.p.i. of Vero E6 cells previously treated with the ARBs, TCID50 was also performed, using four replicate wells by dilution, being read in duplicate.

For this test in Caco-2 and Calu-3 cells, they were cultured in their respective culture medium in 96-well plates three days before the treatment of the cells with the non-cytotoxic concentrations of the ARBs. On treatment day, the medium was collected and replaced by the respective medium containing the respective concentrations of each of the compounds. The cells were maintained for three more days in the presence of the ARBs before being infected with SARS-CoV-2. The infection maintained the parameters of MOI (0.004) and final volume (250 µL/well), with one extra step, in which adsorption was performed by centrifugation at 2,272×g, for one hour at 37°C (Sorvall Legend XT/XF, M-20 rotor, Thermo Scientific™ 75217406/DEL). The cells were then incubated for an additional three days post-infection, when the supernatant was collected for analysis. The remaining procedures and analyses were performed as described for the Vero E6 cell above.

## Results

### Modulation of ACE2 Gene Expression by ARBs in Vero E6 Cells

Initially, we evaluated the modulation of ACE2 and AT1R gene expression in ARBs-treated Vero E6 cells by RT-qPCR. Vero E6 cells are a common model used to evaluate the effect of drugs on coronavirus replication. The ARBs concentrations used in all *in vitro* procedures were defined from the considered non-cytotoxic concentrations by the MTT assays performed in a five-day incubation period in Vero E6 cells and two other SARS-CoV-2 susceptible cells lines (Calu-3 and Caco-2). The suitable non-toxic concentrations were 7 µM for Losartan, Telmisartan, and Valsartan, 15 µM for Azilsartan and Olmesartan Medoxomil, 30 µM for Eprosartan Mesylate, and 60 µM for Irbesartan.

**Figure 1:**
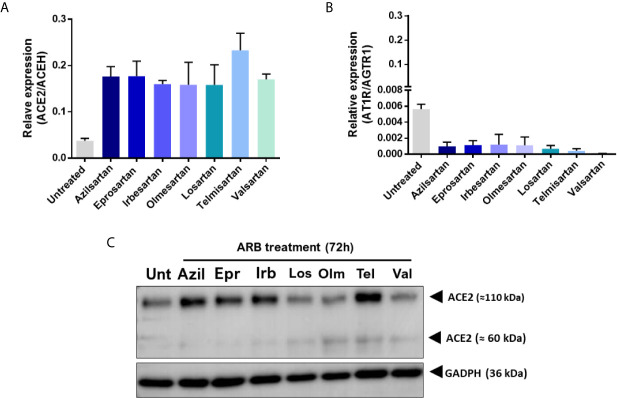
Expression of Angiotensin II receptor type 1 (AT1R) and Angiotensin-converting enzyme 2 (ACE2) mRNAs and proteins by VERO E6 cells following 72 hours incubation with different Angiotensin II Receptor Blockers (ARBs). Non-cytotoxic concentrations of ARBs (Azil, Azilsartan 15µM; Epr, Eprosartan 30µM; Irb, Irbesartan 60µM; Los, losartan 7µM; Olm, Olmesartan 15µM; Tel, Telmisartan 7µM; Val, Valsartan 7µM) were previously defined by the MTT assay and cells were treated with the various ARBs. **(A)** Relative expression of the ACE2/ACEH gene in untreated (Unt) and ARB-treated Vero E6 cells. **(B)** Relative expression of the ATR1/AGTR1 gene in untreated versus ARB-treated Vero E6 cells. **(C)** Expression of ACE2 protein in untreated versus ARB-treated Vero E6 cells (the membrane-anchored ACE2 glycosylated protein that acts as receptor for SARS-CoV-2 is the 110kDa form of the molecule).

Modulation was observed at the transcription of the ACE2 and AT1R genes when these cells were treated for 72 hours with the ARBs (**Figure 1**). While the expression of ACE2 mRNA increased in ARBs-treated Vero E6 (**Figure 1A**), the expression of AT1R mRNA reduced (**Figure 1B**) compared with the untreated control. However, at the protein level, the ACE2 expression increase seems more specific, being stimulated by Azilsartan, Eprosartan, Irbesartan and Telmisartan, representing four of the seven tested ARBs (**Figure 1C**).

When the percentage of positive cells for ACE2 was evaluated by flow cytometry, it was observed that treatment with ARBs does not drastically change the number of cells that are positive for the main receptor of the virus (ACE2). However, not all ARBs have the same effects, and there is a decrease in the number of positive ACE2 cells, particularly under treatment with Losartan and Telmisartan (**Supplementary Figure 1**).

**Figure 2:**
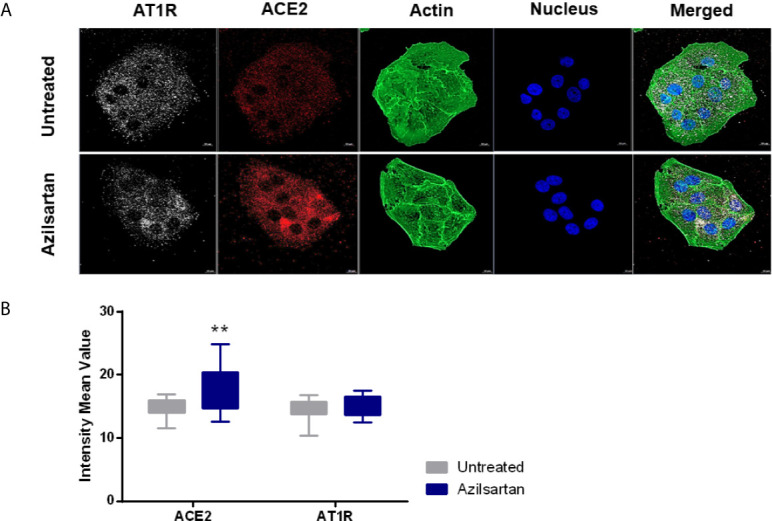
Modulation of cell surface-expressed ATR1 and ACE2 molecules in Vero E6 cells non-infected and treated with Azilsartan (15 µM) for 72 hours by immunofluorescence microscopy. **(A)** The panel presents non-infected cells after incubation with Azilsartan (15 µM) and evaluating the fluorescence corresponding to the ATR1, ACE2, Actin, and the nucleus of the cells. The merge of the images is displayed at the right of the panel. Images were acquired using a confocal microscope (Zeiss LSM 800) with a 63X/1.4 oil objective. **(B)** Quantitative representation of Mean Fluorescence corresponding to ATR1 and ACE2 molecule expression on VERO E6 cells treated or not treated with Azilsartan. **P < 0.01.

When evaluating ACE2 and AT1R protein expression in Vero E6 cells treated with Azilsartan (15 µM) (**Figure 2**), it was possible to observe a wide distribution of AT1R and ACE2 in Vero E6 cells, even in the untreated control (**Figure 2A**), but also a greater fluorescence intensity at the wavelength corresponding to the ACE2 of cells treated with Azilsartan (**Figures 2A, B**). This suggests that the increase in ACE2 expression by Azilsartan is also reflected in cell surface levels. No changes were observed regarding the fluorescence intensity in the wavelength corresponding to AT1R (**Figures 2A, B**).

### Modulation of SARS-CoV-2 Production by ARBs

For the drug testing procedure, Vero E6 cells were selected, as they express an ACE2 receptor compatible with SARS-CoV-2 spike binding, express the ATR1 surface molecule, with which they ARBs will interact (**Figure 2A**), and are known to allow a complete productive viral cycle.

**Figure 3:**
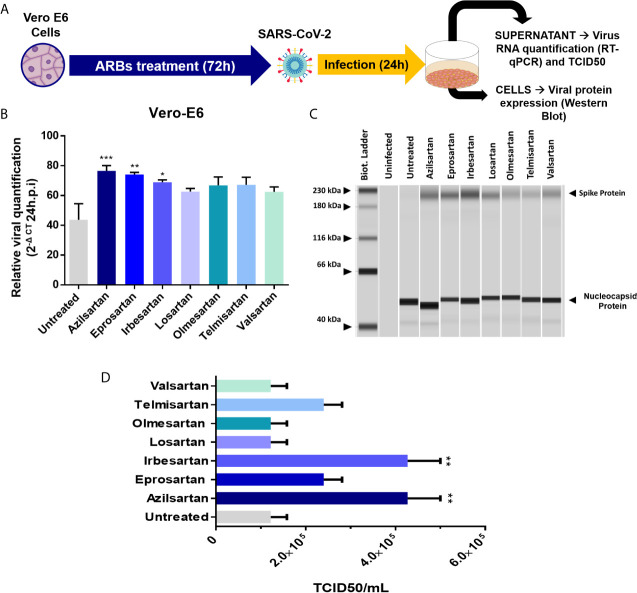
Effects of pre-treating Vero E6 cells in cells with different ARBs on the SARS-CoV-2 production. **(A)** Schematic flow of the analysis: Vero E6 cells were treated with different non-cytotoxic concentrations of ARBs (Azilsartan 15µM; Eprosartan 30µM; Irbesartan 60µM; Losartan 7µM; Olmesartan 15µM; Telmisartan 7µM; Valsartan 7µM) were previously defined by the MTT assay and cells were treated with the different ARBs.) for 72 hours and were subsequently infected and produced virus evaluated by RT-qPCR and Western Blotting 24 hours post-infection (h.p.i.). **(B)** Relative SARS-CoV-2 genome quantification in supernatant of treated and infected Vero E6 cells by RT-qPCR: Relative viral quantification was performed compared to the untreated control (viruses without drugs) using the 2^(–ΔCT)^ method, where ΔCt = {[(Ct 48 h.p.i) treated well] – [mean (Ct 0 h.p.i)]}; **(C)** Viral protein detection in treated and infected Vero E6 cells by Western Blotting performed in JessTM Simple Western system (automated Western immunoblotting), in which every column represents individual runs **(D)** TCID50 (six days post-infection) from the supernatant of the treated with different ARBs Vero E6 cells recovered 24 h.p.i. *P < 0.05; **P < 0.01; ***P < 0.001.

Vero 6 cells were incubated with the different ARBs at previously defined concentrations, for 72 hours before infection with SARS-CoV-2. The relative SARS-CoV-2 replication was evaluated in the cell supernatant, 24 and 48 h.p.i. by RT-qPCR (**Figure 3A**). A significant increase in SARS-CoV-2 replication was observed for Azilsartan, Eprosartan, and Irbesartan treated Vero E6, which represented three of the seven treated groups (**Figure 3B**). This increase was also observed but with less intensity at 48 h.p.i. (data not shown).

**Figure 4:**
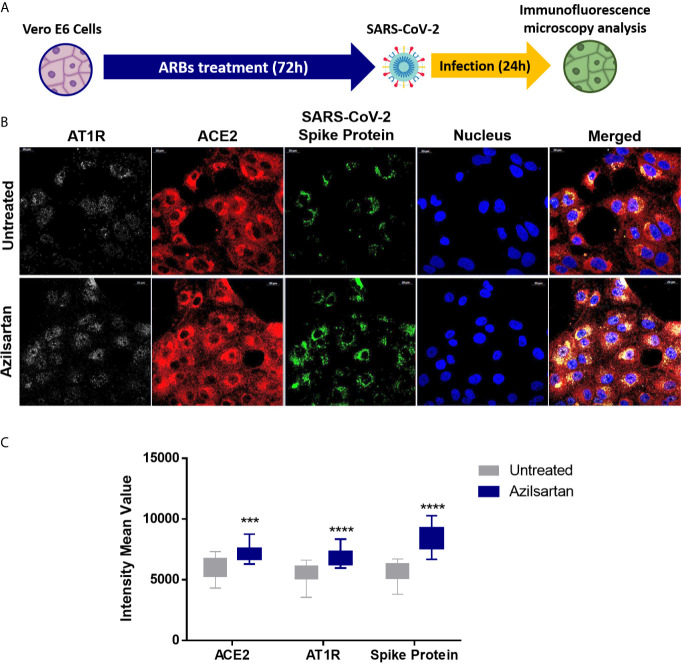
Modulation of cell surface-expressed ATR1 and ACE2 molecules in Vero E6 cells infected with SARS-CoV-2 (24 h.p.i) and treated with Azilsartan (15 µM) for 72 hours by immunofluorescence microscopy. **(A)** Schematic flow of the analysis: Vero E6 cells were treated with various ARBs (the MTT assay previously defined non-cytotoxic concentrations: Azilosartan 15µM; Eprosartan 30µM; Irbesartan 60µM; Losartan 7µM; Olmesartan 15µM; Telmisartan 7µM; Valsartan 7µM) for 72 hours and were subsequently infected for analysis of ACE2 and ATR1 on treated and infected cells, 24 hours post-infection (h.p.i.). **(B)** The panel presents SARS-CoV-2 infected cells after incubation with Azilsartan (15 µM) and evaluation of fluorescence corresponding to the ATR1, ACE2, viral spike protein, and the nucleus of the cells. The merge of the images is displayed at the right of the panel. Images were acquired using a confocal microscope (Zeiss LSM 800) with a 63X/1.4 oil objective. **(C)** Quantitative representation of Mean Fluorescence corresponding to ATR1 and ACE2 molecules expression on VERO E6 cells treated or not treated with Azilsartan and SARS-CoV-2 in the cells. ***P < 0.001; ****P < 0.0001.

Using Western blotting, it was also possible to detect an increase in SARS-CoV-2 spike protein expression in the groups pre-treated with Azilsartan, Eprosartan, and Irbesartan which were infected with SARS-CoV-2 (**Figure 3C**). The TCID50 of the supernatant recovered from ARBs-treated Vero E6 cells 24 h.p.i. also suggests an increase in viral multiplication. The supernatant of cells treated with Azilsartan and Irbesartan presented a TCID50 of 4.27×10^5^ TCID50/mL (**Figure 3D**), which was higher than the untreated control (1.22×10^5^ TCID50/mL) (**Figure 3D**). The groups treated with Eprosartan and Telmisartan displayed a moderated increase, which represents 2.40×10^5^ TCID50/mL (**Figure 3D**), however, this increase was not significant according to statistical analysis. This data suggests that RNA replication and protein production are increased, as is the number of infectious particles released at 24 h.p.i. Increased detection of viral proteins in cells infected with SARS-CoV-2 (24 h.p.i.) treated with Azilsartan and therefore with increased ACE2 expression, was also observed (**Figure 4**).

## Discussion

Since the previous SARS-CoV outbreak, it has been established that the main receptor for this sarbecovirus/beta-coronavirus is the ACE2 receptor, which is also the main cell entry receptor for SARS-CoV-2 (Hoffmann et al., 2020; Zhou et al., 2020). It is understood that the ACE2 protein on the surface of alveolar epithelial cells in the lung allows for respiratory tract infection by SARS-CoV-2. It is assumed that ACE2 levels correlate with a susceptibility to SARS-CoV-2 infection (Devaux et al., 2020). Pre-existing comorbidities, such as respiratory diseases, diabetes and hypertension, in addition to cardiopathy, have been defined as risk factors for a higher lethality of COVID-19 (Chawla et al., 2020; Yang et al., 2020; Zhang et al., 2020).

It is common for individuals with hypertension to use ARBs to control their blood pressure. As its name suggests, this type of medication works by blocking receptors for angiotensin II (AT1 receptor), which has the role of promoting the constriction of blood vessels, increasing blood pressure (Ferrario et al., 2005; Ferrario et al., 2020). These receptors are found in the heart, blood vessels, kidneys, and intestine (Gheblawi et al., 2020; Li et al., 2020; Devaux et al., 2021). Blocking the action of AngII helps to lower blood pressure and prevent damage to the heart and kidneys.

Vero E6 cells were established from kidney tissue from an African green monkey and represent a lineage of mammalian cell lines most often used for isolation and virus production, including SARS-CoV-2 (Naoki et al., 2014; Matsuyama et al., 2020). The ACE2 protein from monkeys showed very moderate polymorphism concerning the ACE2 protein from humans and could interact with the spike protein from SARS-CoV-2 (Devaux et al., 2021; Fenollar et al., 2021). One study suggested that one of the organs in which ACE2 expression levels are higher is the kidneys. The permissiveness of this cell to different viruses, the higher expression of ACE2 (Ren et al., 2006), and the presence of AT1R (**Figure 2A**) make this cell a potential for studying the effects of ARBs on SARS-CoV-2 replication. For this reason, we started by investigating the modulation of the ACE2 and AT1R in Vero E6 cells treated with different ARBs in their respective non-cytotoxic concentration in an AngII-free system. From these analyses, we could observe that ARBs increase the expression of ACE2 mRNA (**Figure 1A**). At the protein level, Azilsartan, Eprosartan, Irbesartan, and Telmisartan induce a higher expression of ACE2 in Vero E6 cells than in untreated cells (**Figure 1C**). While the expression of ACE2 increases, the expression of the receptor to which these blocking molecules bind (AT1R) reduces (**Figure 1B**).

Some animal studies indicate that the use of ARBs leads to the upregulation of ACE2 (Ishiyama et al., 2004; Ferrario et al., 2005; Igase et al., 2008; Soler et al., 2009). In Lewis rats treated for 12 days with Losartan, an increase of cardiac ACE2 mRNA and cardiac ACE2 activity was observed (Ferrario et al., 2005). In this same animal model, Losartan and Olmesartan administered *via* osmotic minipumps for 28 days after coronary artery ligation increased ACE 2 mRNA approximately three-fold (Ishiyama et al., 2004). The proposed mechanism by which these drugs increase the expression of ACE2 associates the inhibitory effect of angiotensin II on Ace2 transcription mediated with the activation of kinase 1 regulated by extracellular signal (ERK1; also known as MAPK3) and ERK2 (also known as MAPK1), after binding to AT1R (Ferrario et al., 2020). This mechanism was revealed by studies in cultured cerebellar or medullary astrocytes, obtained from rats and cardiomyocytes and cardiac fibroblasts from neonatal rats, treated with Losartan and Valsartan (Gallagher et al., 2006; Gallagher et al., 2008).

When it was understood that this modulation of ACE2 expression also occurred in Vero E6 cells, due to data that indicated a rise in the expression of ACE2 mRNA and protein (**Figure 1**), we sought to use immunofluorescence microscopy to investigate whether treatment with ARBs reflected an increase in ACE2 available on the cell surface. Azilsartan appeared to be a potent modulator of the expression of ACE2 in Vero E6 cells, both at the mRNA and protein levels, and therefore was used in this assay. In Vero E6 cells treated for 72 hours with Azilsartan, an increase in the fluorescence average in the wavelength associated with the ACE2 marker was observed (**Figure 2**). This result indicates that the expression of ACE2 would increase on the cell surface, while the superficial AT1R would not change (**Figure 2B**). Interestingly there are significant differences in the ability of ARBs to induce the overexpression of ACE2 once bound to AT1R, since some drugs at 7 µM (e.g. Telmisartan) lead to higher ACE2 increase than others when used at higher concentration (e.g. Irbesartan at 60 µM).

Flow cytometry analysis revealed that neither the number of ACE2-positive cells nor fluorescence intensity changed for most treatments (**Supplementary Figure 1**). In protein expression and in the number of positive ACE2 cells, the divergent results of Losartan are possibly related to its shortest half-life *in vivo* (Oparil, 2000). It is also known that after AngII binding, AT1R triggers ACE2 cleavage and shedding, dependent on the p38 mitogen-activated protein kinases (MAPK) pathway, resulting in reduced cell surface expression (Muslin, 2008; Wu et al., 2020). It has previously been shown that Azilsartan, Candesartan, Losartan, and Telmisartan would have opposite effects on the MAPK pathway, which, consequently, would prevent ACE2 excision (Sekine et al., 2003; Kajiya et al., 2011; Zhong et al., 2011). Therefore, it was understood that number of cells that could be susceptible to SARS-CoV-2 infection remains unchanged, even with quantitative variations in receptor expression. Nevertheless, the increased expression of ACE2 could represent a greater likelihood that the virus would bind to one of these receptors due to the increased availability of receptors on the cell surface, resulting in greater chances of infection success.

Although the adsorption step is essential, since the virus-receptor interaction orchestrates the entry of viruses into the cell, the release of the progeny virions at the end of the replication cycle determines the success of the infection (Maginnis, 2018). Therefore, we chose to evaluate whether Vero E6 cells, already characterised by expressing ACE2 modulated by the ARBs, would change the amount of viral progeny released by the infected cells when pre-treated with these ARBs. The relative production of SARS-CoV-2 was evaluated by RT-qPCR of cellular supernatant, 24 and 48 h.p.i. and after 72 hours of ARB-pre-treatment of Vero E6 cells. The increase in viral RNA expression was significant for three of the ARBs used (Azilsartan, Eprosartan, and Irbesartan) 24 h.p.i. (**Figure 3B**). Interestingly, these three ARBs were also potent positive modulators of ACE2 protein expression (**Figure 1C**).

The estimated complete replication cycle time in Vero E6 cells for SARS-CoV-2 is eight hours (Brahim Belhaouari et al., 2020), so it is challenging to observe significant changes at early stages when using a low MOI. At later stages, however, the saturation of the supernatant by viruses also makes observation difficult to interpret. This difficulty became obvious when a less significant production of SARS-CoV-2 was observed 48 h.p.i. compared to the untreated control at the same time (data not shown). However, in other cell lines, such as Caco-2 and Calu-3, both human-derived cells, this increased effect was not observed (**Supplementary Figure 2**). Previously, the treatment of Calu-3 cells with Losartan and Valsartan did not alter the gene expression of ACE2 (Baba et al., 2020). In this same study, it was suggested that one of the possible reasons for this is the low expression of AT1R, a receptor that interacts with ARBs, in human lung tissues, compared to other organs such as the kidneys (Baba et al., 2020). Therefore, these cells would not be good study models.

When we evaluated the expression of viral proteins inside cells infected with SARS-CoV-2 after treatment with ARBs, these same three compounds (Azilsartan, Eprosartan, and Irbesartan) were shown to induce increased expressions of Spike protein (S) (**Figure 3C**), reinforcing the evidence that these compounds increase the multiplication of SARS-CoV-2. A similar result was observed by immunofluorescence microscopy in cells previously treated with Azilsartan. It is possible to observe the increased detection of ACE2 and SARS-CoV-2 compared to the untreated control (**Figure 4**).

In the viral envelope, glycoprotein S is responsible for the virus attachment to host cells and penetration by membrane fusion (V’kovski et al., 2021). At the same time, the Nucleocapsid (N) of coronaviruses, in addition to playing a structural role, has already been presented as a multifunctional protein, playing a role in the initial RNA synthesis event (McBride et al., 2014). Thus, the higher expression of S protein in the treated groups compared to the untreated groups, may mean infections are established for longer, suggesting that the entry of SARS-CoV-2 may have occurred more quickly due to the increased receptor availability. That notwithstanding, the evaluation of the increased expression of RNA and protein does not necessarily reflect the number of infectious particles produced. Therefore, from the supernatant recovered from the treated and infected cells 24 h.p.i., we performed a TCID50 assay in which it was observed that the ARBs that present modulation of protein ACE2 in Vero E6 (**Figure 1C**) also increased the number of infectious particles at the end of the 24 hour cycle from 1.22×10^5^ TCID50/mL (untreated) to 4.27×10^5^ TCID50/mL (Azilsartan and Irbesartan) (**Figure 3D**).

Our results indicate that Vero E6 cells previously treated for 72 hours with ARBs show a relative increase of ACE2 expression and SARS-CoV-2 production. These results clearly establish a direct relationship between the use of ARBs and viral replication and suggest that when the ARBs bind to the AT1R at the surface of Vero E6 cells, they trigger AT1R-mediated intracellular signals leading to the induction of ACE2 gene expression which likely allows an increase of viral binding, uptake and/or replication. Although the Vero E6 cells model is commonly used to study coronaviruses replication and drugs effect, the relevance of this model in relation to the beneficial use of ARBs to prevent hypertension in patients during periods of high circulation of SARS-CoV-2 remains to be questioned. First, Vero E6 cells were treated with ARBs without adding AngII to the culture medium, while elevated AngII has been reported as a biomarker of severe COVID-19. Second, our experiments were performed in Vero E6 cells, a monkey cellular model. It should be emphasized that the increased SARS-CoV-2 production found in Vero E6 cells was not observed in the preliminary investigations we recently performed on two human cell lines namely Caco-2 (intestinal epithelia origin) and Calu-3 (lung epithelia origin). A wider range of human cell lines should be tested to determine whether it is possible to select an appropriated human cellular model in which ARBs could up-regulated ACE2 expression and SARS-CoV-2 production with the objective to get closer to the conditions that are encountered in human pathophysiology. Based on our current data, we believe that the beneficial use of ARBs to prevent hypertension in patients during periods of high circulation of SARS-CoV-2 should be carefully evaluated.

## Data Availability Statement

The original contributions presented in the study are included in the article/**Supplementary Material**. Further inquiries can be directed to the corresponding author.

## Author Contributions

Conceptualisation: BS and CD. Methodology: BS, CD, and J-PB. Formal analysis: GP, IO, MB, and J-PB. Investigation: GP, IO, RJ, and MB. Data curation: GP, IO, and MB. Writing— original draft preparation: GP and IO. Writing—review and editing: GP, IO, BS, and CD. Supervision: BS and CD. Project administration: BS. All authors contributed to the article and approved the submitted version.

## Funding

This work was supported by the French Government under the “Investments for the Future” programme managed by the National Agency for Research (ANR), Méditerranée-Infection 10-IAHU-03 and was also supported by Région Provence-Alpes-Côte d’Azur and European funding ERDF PRIMMI (European Regional Development Fund - Plateformes de Recherche et d’Innovation Mutualisées Méditerranée Infection).

## Conflict of Interest

CD declares owning shares in the Sanofi and Merck pharmaceutical companies.

The remaining authors declare that the research was conducted in the absence of any commercial or financial relationships that could be construed as a potential conflict of interest.

## Supplementary Material

Supplementary Figure 1 Percentage of ACE2 positive Vero E6 cells after treatment with ARBs. The cells were treated with the ARBs for 72 hours prior to flow cytometry analysis. **(A)** Percentage of ACE2-positive cells after treatment with ARBs; **(B)** Mean Fluorescence Intensity of the cells after treatment with ARBs. Fluorescence intensity was measured using a Canto II cytofluorometer (Becton Dickinson, Biosciences, Le Pont de Claix, France) and the results were analysed using a BD FACSDiva Software v.6.1.3 (Becton, Dickinson and Company, New Jersey, United States). Non-cytotoxic concentrations were previously defined by the MTT assay: Azilsartan 15µM; Eprosartan 30µM; Irbesartan 60µM; Losartan 7µM; Olmesartan 15µM; Telmisartan 7µM; Valsartan 7µM. ***P* < 0.01; ****P* < 0.001.

Supplementary Figure 2 Effects of pre-treating human secondary cells with different ARBs in the SARS-CoV-2 replication. The cells were initially treated with the drugs for 72 hours before infection and incubated in their presence for additional three days post-infection. The supernatant was collected 72 h.p.i. for RNA extraction and RT-qPCR. Relative viral quantification was performed and compared to the untreated control using the 2(–ΔCT) method. **(A)** Relative viral genome quantification of SARS-CoV-2 in the supernatant of treated and infected Caco-2 cells. **(B)** Relative SARS-CoV-2 genome quantification in the supernatant of treated and infected Calu-3 cells. Non-cytotoxic concentrations were previously defined by MTT assay. **P <* 0.05; *****P* < 0.0001.
