# Supplementary figures and images for: Angiotensin II Receptor Blockers (ARBs Antihypertensive Agents) Increase Replication of SARS-CoV-2 in Vero E6 Cells
Source: Front Cell Infect Microbiol. 2021 Jun 11;11:639177. doi: 10.3389/fcimb.2021.639177 (PMC8231006; doi:10.3389/fcimb.2021.639177)

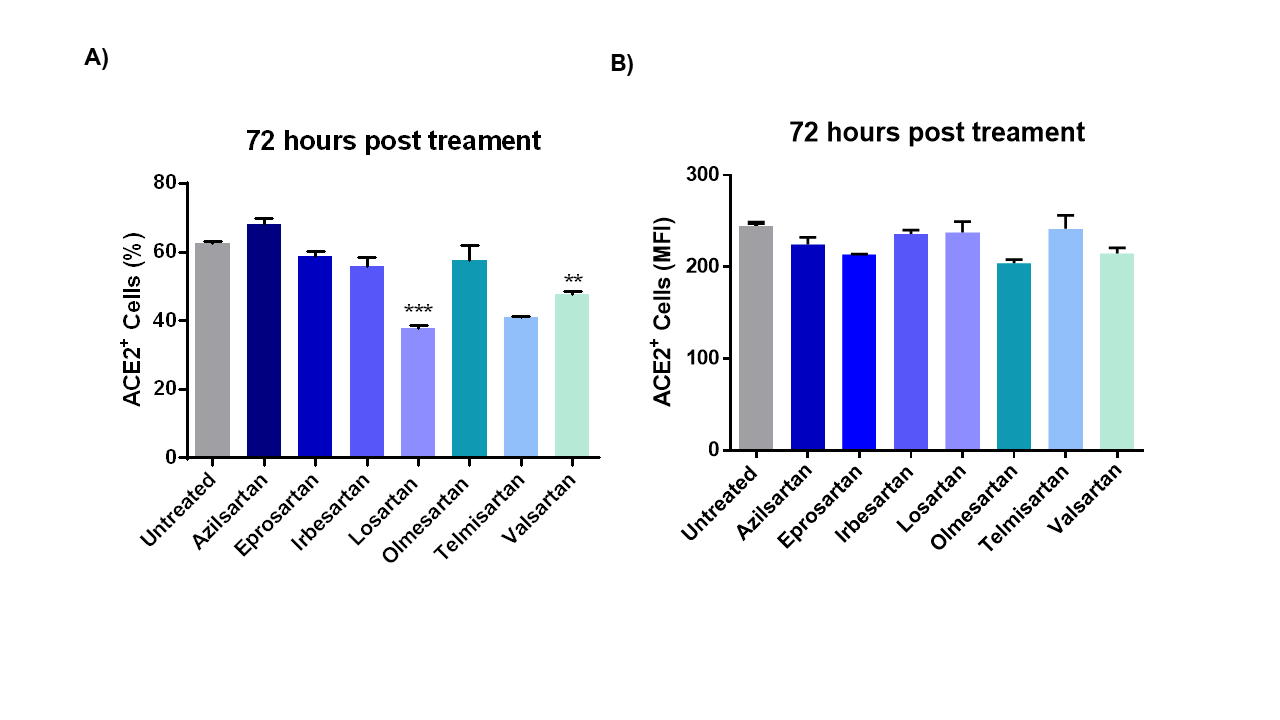

Supplement: Supplementary Figure 1 — Percentage of ACE2 positive Vero E6 cells after treatment with ARBs. The cells were treated with the ARBs for 72 hours prior to flow cytometry analysis. (A) Percentage of ACE2-positive cells after treatment with ARBs; (B) Mean Fluorescence Intensity of the cells after treatment with ARBs. Fluorescence intensity was measured using a Canto II cytofluorometer (Becton Dickinson, Biosciences, Le Pont de Claix, France) and the results were analysed using a BD FACSDiva Software v.6.1.3 (Becton, Dickinson and Company, New Jersey, United States). Non-cytotoxic concentrations were previously defined by the MTT assay: Azilsartan 15µM; Eprosartan 30µM; Irbesartan 60µM; Losartan 7µM; Olmesartan 15µM; Telmisartan 7µM; Valsartan 7µM. **P < 0.01; ***P < 0.001. [file Image_1.tif]

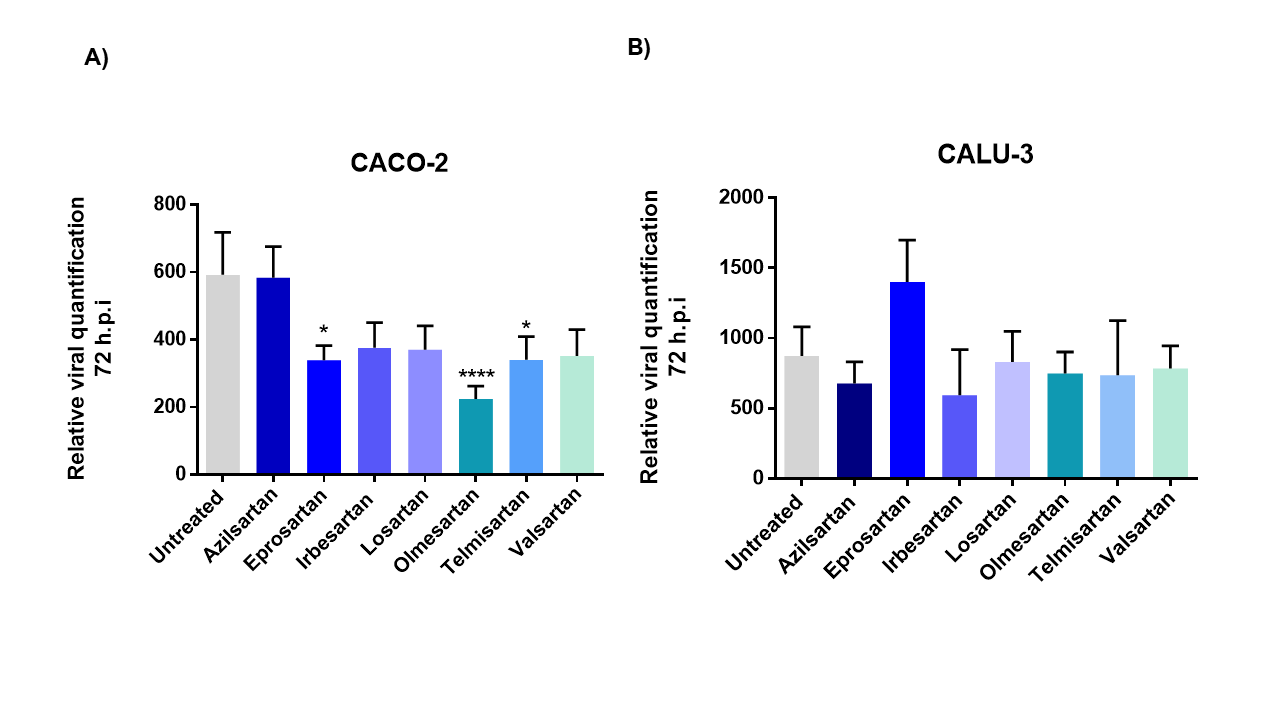

Supplement: Supplementary Figure 2 — Effects of pre-treating human secondary cells with different ARBs in the SARS-CoV-2 replication. The cells were initially treated with the drugs for 72 hours before infection and incubated in their presence for additional three days post-infection. The supernatant was collected 72 h.p.i. for RNA extraction and RT-qPCR. Relative viral quantification was performed and compared to the untreated control using the 2(–ΔCT) method. (A) Relative viral genome quantification of SARS-CoV-2 in the supernatant of treated and infected Caco-2 cells. (B) Relative SARS-CoV-2 genome quantification in the supernatant of treated and infected Calu-3 cells. Non-cytotoxic concentrations were previously defined by MTT assay. *P < 0.05; ****P < 0.0001. [file Image_2.tif]
